# Matrix Metalloproteinase Genes Are Associated with Breast Cancer Risk and Survival: The Breast Cancer Health Disparities Study

**DOI:** 10.1371/journal.pone.0063165

**Published:** 2013-05-16

**Authors:** Martha L. Slattery, Esther John, Gabriela Torres-Mejia, Mariana Stern, Abbie Lundgreen, Lisa Hines, Anna Giuliano, Kathy Baumgartner, Jennifer Herrick, Roger K. Wolff

**Affiliations:** 1 Department of Medicine, University of Utah, Salt Lake City, Utah, United States of America; 2 Cancer Prevention Institute of California, Fremont, California, United States of America; 3 Division of Epidemiology, Department of Health Research and Policy and Stanford Cancer Institute, Stanford University School of Medicine, Stanford, California, United States of America; 4 Instituto Nacional de Salud Pública, Centro de Investigación en Salud Poblacional, Ahuacatitlán, Cuernavaca, Morelos, México; 5 Department of Preventive Medicine, Keck School of Medicine of USC, University of Southern California, Los Angeles, California, United States of America; 6 Department of Biology, University of Colorado at Colorado Springs, Colorado Springs, Colorado, United States of America; 7 Moffitt Cancer Center and Research Institute, Cancer Prevention and Control, Tampa, Florida, United States of America; 8 Department of Epidemiology and Population Health, School of Public Health & Information Sciences, University of Louisville, Louisville, Kentucky, United States of America; Ohio State University Medical Center, United States of America

## Abstract

Matrix metalloproteinases (MMPs) contribute to cancer through their involvement in cancer invasion and metastasis. We evaluated genetic variation in *MMP1* (9 SNPs), *MMP2* (8 SNPs), *MMP3* (4 SNPs), and *MMP9* (3 SNPs) and breast cancer risk among Hispanic (2111 cases, 2597 controls) and non-Hispanic white (NHW) (1481 cases, 1586 controls) women in the Breast Cancer Health Disparities Study. Ancestral informative markers (n = 104) were assessed to determine Native American (NA) ancestry. *MMP1* [4 single nucleotide polymorphisms (SNPs)] and *MMP2* (2 SNPs) were associated with breast cancer overall. *MMP1* rs996999 had strongest associations among women with the most NA ancestry (OR 1.61,95% CI 1.09,2.40) as did *MMP3* rs650108 (OR 1.36, 95% CI 1.05,1.75) and *MMP9* rs3787268 (OR 1.52, 95% CI 1.09,2.13). The adaptive rank truncated product (ARTP) showed a significant pathway p_artp_ value of 0.04, with a stronger association among women with the most NA ancestry (p_artp_ = 0.02). Significant pathway genes using the ARTP were *MMP1* for all women (p_artp_ = 0.02) and *MMP9* for women with the most NA ancestry (p_artp_ = 0.024); *MMP2* was borderline significant overall (p_artp_ = 0.06) and *MMP1* and *MMP3* were borderline significant for women with the most NA ancestry (p_artp_ = 0.07 and 0.06 respectively). *MMP1* and *MMP2* were associated with ER+/PR+ and ER+/PR-tumors; *MMP3* and *MMP9* were associated with ER−/PR− tumors. The pathway was highly significant with survival (p_artp_ = 0.0041) with *MMP2* having the strongest gene association (p_artp_ = 0.0007). Our findings suggest that genetic variation in *MMP* genes influence breast cancer development and survival in this genetically admixed population.

## Introduction

Matrix metalloproteinase (MMP) plays an important role in cancer progression by degrading extracellular matrix and basement membrane and are the main proteolytic enzymes involved in cancer invasion and metastasis [1]. MMPs are involved in normal physiological processes required for development and morphogenesis; a loss of control of MMPs can result in pathological processes including inflammation, angiogenesis, and cellular proliferation that are central to diseases such as cancer. MMPs are categorized into five groups based on their structure and substrate specificity: collagenases, gelatinases, stromelysins, matrilysins and membrane MMPs [2]. Collagenases include MMP-1, MMP-8, MMP-13, and MMP-18; MMP-1 is one of the most widely expressed MMP and can degrade type I, II, and III collagens. Gelatinases A (MMP-2) and B (MMP-9) digest gelatins or denatured collagens and are two of the most widely studied MMPs in cancer. MMP-9 is one of the most complex members of the MMP family and expression of MMP-9 is up-regulated in breast cancer [3]. Stromelysins include MMP-3 and MMP-10. MMP-3 has a proteolytic efficiency that is higher than MMP-10 and activates a number of proMMPs. Matrilysins include MMP-7 and MMP-26 and process cell surface molecules.

Polymorphisms in the *MMP1*, *MMP2*, *MMP3*, and *MMP9* genes have been examined in studies evaluating cancer metastasis [3], [4] and functional polymorphisms have been identified for these genes. Polymorphisms in *MMP1* −1607, *MMP3* −1171 and *MMP9* −1562 have been associated with general cancer metastasis in a large meta-analysis [3]. *MMP1* −1607 has shown stronger associations among individuals with more European ancestry [3]. Although *MMP2* −1306 was not associated with overall cancer metastasis in that study, other studies have shown this polymorphism to be associated with tumor size, estrogen receptor status and survival [5]. Polymorphisms in *MMP2* have been associated with breast cancer risk specifically in the Shanghai Breast Cancer Study, a large case-control study of over 6000 Chinese women [6], and in a small study of 90 cases and 96 controls in Mexico [7]. Polymorphisms in *MMP1* and *MMP3* were not associated with breast cancer risk in the Shanghai Breast Cancer Study [8]

In this study we evaluated genetic variation in *MMP1*, *MMP2*, *MMP3*, and *MMP9* using data from a large collaborative case-control study of breast cancer in Hispanic and non-Hispanic white women (NHW) from the United States and Mexico. It is of interest to evaluate these genes and their association with breast cancer among these populations because of the observed ethnic differences in breast cancer incidence and survival rates [9]. While differences in screening and lifestyle factors likely contribute to racial/ethnic disparities in breast cancer, differences in genetic susceptibility are also likely to play a significant role. Although MMPs are important components in cancer invasiveness, few studies have evaluated the role of *MMP* polymorphisms in breast cancer risk and survival taking into account tumor characteristics. In this study we used a comprehensive tagSNP approach to evaluate associations with breast cancer risk and survival, taking into account genetic admixture, menopausal status, estrogen receptor (ER) and progesterone receptor (PR) tumor status, tumor grade, and disease stage.

## Methods

The Breast Cancer Health Disparities Study includes participants from three population-based case-control studies, the 4-Corner's Breast Cancer Study, the Mexico Breast Cancer Study, and the San Francisco Bay Area Breast Cancer Study [10] who completed an in-person interview and who had a blood or mouthwash sample available for DNA extraction. In the 4 Corner's Breast Cancer Study, participants were between 25 and 79 years of age with a histological confirmed diagnosis of *in situ* (n = 341) or invasive (n = 1492) cancer between October 1999 and May 2004; controls were selected from the target populations of cases living in Arizona, Colorado, New Mexico, and Utah and were frequency matched to cases on ethnicity and 5-year age distribution [11]. Participants from the Mexico Breast Cancer Study were between 28 and 74 years of age. Eligible cases in Mexico were women diagnosed with either a new histologically confirmed *in situ* or invasive breast cancer between January 2004 and December 2007 at 12 participating hospitals from three main health care systems; controls were randomly selected from the catchment area of the 12 participating hospitals using a probabilistic multi-stage design. The San Francisco Bay Area Breast Cancer Study included women aged 35 to 79 years from the San Francisco Bay Area diagnosed with a first primary histologically confirmed invasive breast cancer between April 1995 and April 2002; controls were identified by random-digit dialing (RDD) and frequency-matched to cases based on the expected race/ethnicity and 5-year age distribution [12], [13]. Since associations did not differ when including or excluding *in situ* cases, the results presented include both. All participants signed informed written consent prior to participation; this study was approved by the Institutional Review Board for Human Subjects at the University of Utah, Comisión de ética, and Institutional Review Board of the Cancer Prevention Institute of California.

### Data Harmonization

Data were harmonized across all study centers and questionnaires as previously described [10]. Women were classified as either pre-menopausal or post-menopausal based on responses to questions on menstrual history. Women who reported still having periods during the referent year (defined as the year before diagnosis for cases or before selection into the study for controls) were classified as pre-menopausal. Center-specific definitions were used to define post-menopausal women. Women were classified as post-menopausal if they reported either a natural menopause or If they reported taking hormone therapy (HT) and were still having periods or were at or above the 95th percentile of age for those who reported having a natural menopause (i.e., ≥12 months since their last period. This age at menopause was site specific by ethnicity: 58 for NHW and 56 for Hispanic women from the 4-Corner's Breast Cancer Study; 54 for the Mexico Breast Cancer Study; and 55 for NHW and 56 for Hispanic women from the San Francisco Bay Area Breast Cancer Study.

### Genetic Data

DNA was extracted from either whole blood or mouthwash samples; 7287 blood-derived and 634 mouthwash-derived samples were available. Whole Genome Amplification (WGA) was applied to the mouthwash-derived DNA samples prior to genotyping. A tagSNP approach was used to characterize variation across candidate genes. TagSNPs were selected using the following parameters: linkage disequilibrium (LD) blocks were defined using a Caucasian LD map and an r^2^ = 0.8; minor allele frequency (MAF) >0.1; range = −1500 bps from the initiation codon to +1500 bps from the termination codon; and 1 SNP/LD bin. Additionally, 104 Ancestral Informative Markers (AIMs) were used to distinguish European and Native American ancestry in the study population [10]. All markers were genotyped using a multiplexed bead array assay format based on GoldenGate chemistry (Illumina, San Diego, California). A genotyping call rate of 99.93% was attained (99.65% for WGA samples). We included 132 blinded internal replicates representing 1.6% of the sample set. The duplicate concordance rate was 99.996% as determined by 193,297 matching genotypes among sample pairs. In the current analysis we evaluated *MMP1* (9 SNPs), *MMP2* (8 SNPs), *MMP3* (4 SNPs), and *MMP9* (3 SNPs). A description of these genes and all SNPs is shown in Table 1.

**Table 1:** Summary of *MMP* genes and SNPs assessed.

		Chromosome		Major/Minor	MAF^1^		FDR HWE^2^ P	
Gene	Aliases	Location	dbSNP ID	Allele	NHW^3^	HISP/NA^3^	NHW	HISP/NA
*MMP1*	CLG, CLGN	11q22.3	rs5854	C/T	0.38	0.22	0.96	0.52
			rs17293823	G/A	0.13	0.08	1.00	0.71
			rs996999	C/T	0.19	0.26	0.98	0.46
			rs17293761	C/T	0.08	0.07	0.96	0.80
			rs7945189	C/T	0.10	0.05	0.96	0.13
			rs7125062	T/C	0.27	0.43	0.98	0.83
			rs470358	C/T	0.39	0.45	0.96	0.85
			rs475007	A/T	0.45	0.45	0.96	0.52
			rs1144393	T/C	0.40	0.20	0.97	0.27
*MMP2*	CLG4, CLG4A,	16q13-q21	rs243839	A/G	0.18	0.28	0.98	0.40
	MMP-II, MONA		rs1477017	A/G	0.35	0.44	0.96	0.95
	TBE-1		rs1992116	C/T	0.44	0.41	0.78	0.95
			rs243836	G/A	0.49	0.43	0.62	0.47
			rs243845	C/T	0.39	0.31	0.96	0.68
			rs243865	C/T	0.24	0.21	0.96	0.93
			rs11639960	A/G	0.34	0.35	0.97	0.46
			rs11541998	C/G	0.11	0.06	0.98	0.19
*MMP3*	CHDS6, MGC126102,	11q22.3	rs520540	G/A	0.52	0.32	0.62	0.29
	MGC126103, MGC126104		rs569444	G/A	0.12	0.08	0.96	0.38
	MMP-3, SL-1, STMY		rs650108	G/A	0.26	0.53	0.96	0.20
	STMY1STR1		rs522616	A/G	0.21	0.46	1.00	0.22
*MMP9*	CLG4B, GELB,	20q11.2-q13.1	rs3918261	A/G	0.15	0.08	0.98	0.33
	MANDP2, MMP-9		rs3918249	T/C	0.38	0.24	0.98	0.21
			rs3787268	G/A	0.22	0.12	0.98	0.12

1 Minor Allele Frequency (MAF).

2 Hardy Weinberg Equilibrium (HWE).

3 NHW (Non-Hispanic White); Hisp/NA (Hispanic/Native American).

#### Tumor Characteristics and Survival

Cancer registries in Utah, Colorado, Arizona, New Mexico, and California provided information on stage at diagnosis, months of survival after diagnosis, cause of death, and estrogen receptor (ER) and progesterone receptor (PR) status. Information on ER and PR status of tumors was available for 1019 (69%) NHW and 977 (75%) Hispanic cases. Survival information, tumor grade, and stage at diagnosis were not available for cases from Mexico.

### Statistical Methods

The program STRUCTURE was used to compute individual ancestry for each study participant assuming two founding populations [14], [15]. A three-founding population model was assessed but did not fit the population structure with the same level of repeatability and correlation among runs as the two-founding population model. Participants were classified by level of percent Native American ancestry. Assessment across categories of ancestry was done using cut-points based on the distribution of genetic ancestry in the control population with the goal of creating distinct ancestry groups that had sufficient power to assess associations. Three strata, 0–28%, 29 to 70%, and 71 to 100%, were used to evaluate associations by level of Native American (NA) ancestry. Genetic ancestry was used as a continuous variable when included in the models to adjust for possible confounding.

All statistical analyses were performed using SAS version 9.3 (SAS Institute, Cary, NC). Genes and SNPs were assessed for their association with breast cancer risk for all women and by strata of genetic ancestry, ER/PR status, and menopausal status. Logistic regression models were used to estimate odds ratios (OR) and 95% confidence intervals (CI) for breast cancer risk associated with SNPs, adjusting for age, study center, genetic ancestry, reference year BMI, and parity. Associations with SNPs were assessed assuming a co-dominant model. Based on the initial assessment, SNPs which appeared to have a dominant or recessive mode of inheritance were evaluated with those inheritance models in subsequent analyses.

The p values used to adjust for multiple comparisons were based on Wald chi-square test statistics comparing the homozygote variant to the wildtype for additive/co-dominant models, the homozygote variant/heterozygote to the wildtype for dominant models, and the homozygote variant to the wildtype/heterozygote for recessive models. They were adjusted for multiple comparisons taking into account tagSNPs within the gene using the step-down Bonferroni correction (i.e., Holm method). This method of correction for multiple comparisons is very conservative, especially for correlated variables such as SNPs within a gene. To take into account the correlated nature of the data, we determined the effective number of independent SNPs using the SNP spectral decomposition method proposed by Nyholt [16] and modified by Li and Ji [17]. Raw p values that were unadjusted for multiple comparisons are reported since we assessed hypothesized genes in a candidate pathway; we also report adjusted p values taking into account the number of SNPs being assessed.

Survival months were calculated based on month and year of diagnosis and month and year of death or date of last contact. Associations between SNPs and risk of dying of breast cancer among primary invasive cases were evaluated using Cox proportional hazards models to obtain multivariate hazard ratios (HR) and 95% CI for all women and by admixture strata. Since survival data were not available for the Mexico study site, the upper two admixture strata were combined to evaluate survival. Individuals were censored when they died of causes other than breast cancer or were lost to follow-up. In addition to the minimal adjustments for age, study center, genetic ancestry, referent year BMI, and parity, models were also adjusted for SEER summary stage to estimate HRs. Generalized logit models were used to assess associations between SNPs and both tumor stage and grade. In these models, the comparison group for calculation of ORs for tumor stage was localized/in situ while associations with grade were well differentiated tumors.

Interactions between genetic variants and genetic ancestry and genetic variants and menopausal status were assessed using one degree of freedom (1-df) Wald chi-square tests. Differences in risk by ER/PR status were estimated using Wald chi-square tests from generalized logit model. Interactions between genetic variants and genetic ancestry with survival were assessed using p values from Wald chi-square tests.

Haplotypes were developed to help define risk associated with genes. SNPs were selected based on their individual significance overall or within a genetic ancestry group. Haplotypes were estimated using the expectation maximization algorithm. Per-copy and copy number haplotype risk estimates were obtained using logistic regression and adjusted for age, study center, genetic admixture, BMI in referent year, and parity. We focused on haplotypes with a frequency of ≥0.05 in reporting results since those with lower frequency were generally imprecise.

We used the adaptive rank truncated product (ARTP) method that utilizes a highly efficient permutation algorithm to determine the significance of association of each gene and of the MMP pathway with breast cancer overall, by admixture, and by ER/PR strata. To estimate the ARTP for survival we categorized the outcome as death from breast cancer versus alive to approximate the associations using the proportional hazard models. The gene p values were generated using the ARTP package in R, permuting case-control status 10,000 times while adjusting for age, reference year BMI, and genetic admixture [18], [19]. Models approximating survival also were adjusted for SEER stage. We report both pathway and gene p values (p_artp_)

## Results

The majority of breast cancer cases were Hispanic, under 60 years of age, and post-menopausal (Table 2). Among U.S. cases, most tumors were ER+/PR+ followed by ER−/PR− tumors, accounting for 18.4% of NHW and 23.4% of Hispanic cases (note ER and PR status was not available for Mexican women). The majority of women who self-reported being NHW were estimated as having low Native American Ancestry (99.5% of controls). U.S. women who self-reported being Hispanic where mostly divided between those with intermediate Native American ancestry (64.9% of controls) and high Native American ancestry (24.4% of controls).

**Table 2:** Description of Study Population by Race/Ethnicity.

	Non-Hispanic White				U. S. Hispanic or Mexican			
	Controls		Cases		Controls		Cases	
	N	%	N	%	N	%	N	%
Total	1586	37.9	1481	41.2	2597	62.1	2111	58.8
Study Site								
4 Corner's	1322	83.4	1227	82.8	723	27.8	597	28.3
Mexico City	0	0	0	0	994	38.3	816	38.7
San Francisco Bay Area	264	16.6	254	17.2	880	33.9	698	33.1
Age (years)								
<40	116	7.3	89	6	311	12	200	9.5
40–49	408	25.7	409	27.6	831	32	713	33.8
50–59	409	25.8	413	27.9	756	29.1	617	29.2
60–69	350	22.1	361	24.4	526	20.3	430	20.4
≥70	303	19.1	209	14.1	173	6.7	151	7.2
Mean	56.6		56		52.3		52.7	
Menopausal Status								
Pre-menopausal	494	31.5	489	33.5	1027	40.7	836	40.9
Post-menopausal	1076	68.5	970	66.5	1499	59.3	1210	59.1
Estimated Native American Ancestry								
Low (0–28%)	1578	99.5	1472	99.4	278	10.7	275	13
Intermediate (29–70%)	7	0.4	7	0.5	1686	64.9	1393	66
High (71–100%)	1	0.1	2	0.1	633	24.4	443	21
ER/PR Status^1^								
ER+/PR+	NA		695	68.2	NA		605	61.9
ER+/PR−	NA		121	11.9	NA		115	11.8
ER−/PR+	NA		15	1.5	NA		28	2.9
ER−/PR−	NA		188	18.4	NA		229	23.4

1 Tumor information unavailable for the Mexico study site.

### MMP Polymorphisms and breast cancer risk

Among all women combined, *MMP1* and *MMP2* were associated with breast cancer risk (Table 3). When stratifying by percent Native American ancestry, *MMP3* and *MMP9* were associated with breast cancer risk among women with more Native American ancestry only; the p for interaction with *MMP9* remained statistically significant after adjustment for multiple comparisons (p_adj_ = 0.002) (Table 3). Utilization of the ARTP method to determine pathway and gene significance showed that overall the *MMP* pathway was significantly associated with breast cancer risk (p_artp_ = 0.04) with the strongest association observed for women with the most Native American ancestry (p_artp_ = 0.02). *MMP1* was significant overall (p_artp_ = 0.02) and *MMP2* was borderline significant (p_artp_ = 0.06). Among women with the most Native American ancestry, *MMP1* and *MMP3* were of borderline significance (p_artp_ = 0.07 and 0.06 respectively) whereas *MMP9* was statistically significant among this group of women (p_artp_ = 0.024).

**Table 3:** Associations between *MMP* genes and breast cancer risk by Native American Ancestry.

	Everyone				0–28% Native American Ancestry				29–70% Native American Ancestry				71–100% Native American Ancestry				
	Controls	Cases			Controls	Cases			Controls	Cases			Controls	Cases			Interaction
	N	N	OR^1^	(95% CI)	N	N	OR	(95% CI)	N	N	OR	(95% CI)	N	N	OR	(95% CI)	P-value
*MMP1* (rs5854)																	0.20
CC/CT	3785	3291	1.00		1584	1548	1.00		1587	1316	1.00		614	427	1.00		
TT	365	278	0.82	(0.69, 0.97)	258	194	0.78	(0.63, 0.95)	92	74	0.95	(0.69, 1.31)	15	10	0.88	(0.38, 2.00)	
P-value (raw; adjusted)^2^			0.018, 0.14				0.01, 0.09				0.75, 1.00				0.76, 1.00		
*MMP1* (rs996999)																	0.13
CC/CT	3933	3354	1.00		1776	1677	1.00		1585	1299	1.00		572	378	1.00		
TT	216	213	1.23	(1.01, 1.50)	65	64	1.03	(0.73, 1.47)	94	90	1.23	(0.91, 1.66)	57	59	1.61	(1.09, 2.40)	
P-value (raw; adjusted)			0.039, 0.21				0.86, 0.86				0.18, 1.00				0.018, 0.14		
*MMP1* (rs7125062)																	0.38
TT/TC	3547	3030	1.00		1696	1607	1.00		1396	1127	1.00		455	296	1.00		
CC	599	539	1.15	(1.01, 1.32)	143	135	0.99	(0.78, 1.27)	283	263	1.21	(1.00, 1.47)	173	141	1.36	(1.03, 1.79)	
P-value (raw; adjusted)			0.03, 0.21				0.96, 0.96				0.045, 0.36				0.029, 0.20		
*MMP1* (rs1144393)																	0.32
TT	2240	1924	1.00		695	696	1.00		1043	869	1.00		502	359	1.00		
TC	1536	1336	0.94	(0.85, 1.04)	858	806	0.94	(0.82, 1.09)	557	461	0.97	(0.83, 1.14)	121	69	0.76	(0.54, 1.06)	
CC	373	305	0.83	(0.70, 0.98)	288	237	0.83	(0.68, 1.02)	79	59	0.82	(0.58, 1.18)	6	9	1.69	(0.58, 4.87)	
P-value (raw; adjusted)			0.03, 0.21				0.07, 0.36				0.29, 1.00				0.33, 1.00		
*MMP2* (rs243845)																	0.45
CC/CT	3646	3183	1.00		1563	1498	1.00		1490	1269	1.00		593	416	1.00		
TT	504	386	0.84	(0.73, 0.97)	279	244	0.91	(0.76, 1.10)	189	121	0.74	(0.58, 0.94)	36	21	0.82	(0.47, 1.43)	
P-value (raw; adjusted)			0.015, 0.08				0.34, 1.00				0.016, 0.08				0.48, 1.00		
*MMP2* (rs11541998)																	0.40
CC	3547	2957	1.00		1486	1364	1.00		1456	1180	1.00		605	413	1.00		
CG/GG	600	610	1.16	(1.02, 1.31)	356	376	1.14	(0.97, 1.35)	220	210	1.12	(0.91, 1.38)	24	24	1.19	(0.65, 2.17)	
P-value (raw; adjusted)			0.02,0.08				0.11, 0.55				0.29, 0.60				0.58, 1.00		
*MMP3* (rs650108)																	0.61
GG/GA	3282	2879	1.00		1709	1619	1.00		1238	1052	1.00		335	208	1.00		
AA	868	689	1.01	(0.89, 1.13)	133	122	0.96	(0.74, 1.24)	441	338	0.94	(0.80, 1.11)	294	229	1.36	(1.05, 1.75)	
P-value (raw; adjusted)			0.936, 1.000				0.77, 0.77				0.48, 1.00				0.019, 0.06		
*MMP9* (rs3787268)																	<.001
GG	2930	2479	1.00		1129	1107	1.00		1258	1025	1.00		543	347	1.00		
GA/AA	1202	1074	1.00	(0.91, 1.11)	712	632	0.91	(0.79, 1.04)	405	355	1.06	(0.89, 1.25)	85	87	1.52	(1.09, 2.13)	
P-value (raw; adjusted)			0.96, 0.96				0.15, 0.24				0.53, 0.79				0.015, 0.037		

1 Adjusted for age, study center, reference year BMI, parity and genetic admixture.

2 P values are given for raw and adjusted for multiple comparisons; includes only SNPs with significant effects.

Only *MMP2* rs243845 showed significant differences in associations by menopausal status (data not shown). *MMP2* rs243845 was significantly associated with breast cancer risk among post-menopausal women only (OR 0.73, 95% CI 0.61,0.88 for TT vs CC genotypes; adjusted p for interaction = 0.036).

Evaluation of haplotypes for *MMP1* and *MMP2* showed statistically significant haplotype associations using both the additive model (Table S1) and the copy number of haplotype (data not shown in supplement). Associations with haplotypes were modest. For *MMP1* the number of copies of the haplotype was important, most likely because of the significance observed among SNPs with the recessive model. Having two copies of the *MMP1* CTCT haplotype was associated with increased risk compared to zero copies (OR 1.31 95% CI 1.04, 1.64 p = 0.022 for 2 vs. 0 copies) for the entire population , and with the strongest association found in women with the highest Native American ancestry (OR 1.77, 95% CI 1.16, 2.69; p = 0.008). Similarly, the converse of the haplotype *MMP1* TCTC was inversely associated with breast cancer risk when looking at copy number for all women (OR 0.65, 95% CI 0.45,0.95 p = 0.027) and among women with the least Native American ancestry specifically (OR 0.60, 95% CI 0.40,0.91 p = 0.015).

Polymorphisms in all of the *MMP* genes were associated with ER/PR tumor status prior to adjustment for multiple comparisons (Table 4). *MMP1* rs5854, rs470358, and rs1144393 and *MMP2* rs1477017 and rs243845 were associated with ER+/PR+ tumors, however only rs5854 and rs1144393 showed significant p values for heterogeneity (p = 0.03 and 0.04 respectively) and rs1144393 remained significant after multiple comparison adjustment. *MMP1* rs5854 and *MMP2* rs1477017 and rs243836 also were associated with ER+/PR− tumors (adjusted p values = 0.03, 0.06, and 0.04 respectively). Only *MMP1* rs7125062 was associated with ER−/PR+ tumors prior to multiple comparison adjustment. *MMP2* rs243839 and *MMP3* rs650108 and *MMP9* rs3918261 and rs3918249 were associated with ER−/PR− tumors prior to multiple comparison adjustment. Assessment of haplotypes for *MMP1*, *MMP2*, and *MMP9* showed several statistically significant associations (see Table S2). The TTCC *MMP1* genotype was associated with reduced risk of ER+/PR+ and ER+/PR− tumors. The most common *MMP2* haplotype, AGGC was associated with increased risk of ER+/PR− tumors. The rare *MMP9* GC haplotype was associated with ER+/PR− tumors whereas the AT and GC haplotypes were associated with ER−/PR− tumors. P values for ARTP showed the pathway significantly associated with both ER+/PR+ and ER−/PR− tumors (p_artp_ = 0.032 and 0.034 respectively); *MMP1* was most significantly associated with ER+/PR+ tumors (p_artp_ = 0.013), while both *MMP3* and *MMP9* were associated with ER−/PR− tumors (p_artp_ = 0.04 and 0.046 respectively).

**Table 4:** Associations between *MMP* genes and ER and PR tumor status.

	Controls	ER+/PR+			ER+/PR−			ER−/PR+			ER−/PR−			Interaction p value
	N	N	OR^1^	(95% CI)	N	OR	(95% CI)	N	OR	(95% CI)	N	OR	(95% CI)	
*MMP1* (rs5854)														
CC	1535	663	1.00		124	1.00		21	1.00		206	1.00		0.028
CT	1303	516	0.87	(0.76, 1.00)	100	0.91	(0.69, 1.21)	16	0.93	(0.48, 1.80)	171	0.97	(0.78, 1.21)	
TT	328	119	0.79	(0.62, 0.99)	11	0.40	(0.21, 0.76)	6	1.47	(0.58, 3.77)	38	0.88	(0.61, 1.28)	
P-value (raw; adjusted)^2^			0.045, 0.23			0.005, 0.03			0.42, 1.00			0.50, 1.00		
*MMP1* (rs7125062)														
TT	1411	577	1.00		105	1.00		26	1.00		190	1.00		0.18
TC/CC	1752	721	1.05	(0.92, 1.19)	130	1.01	(0.77, 1.33)	17	0.48	(0.26, 0.90)	225	0.93	(0.76, 1.15)	
P-value (raw; adjusted)			0.51, 0.72			0.92, 1.00			0.023, 0.16			0.51, 1.00		
*MMP1* (rs470358)														
CC	1092	415	1.00		65	1.00		16	1.00		141	1.00		0.15
CT	1544	638	1.08	(0.94, 1.26)	133	1.43	(1.05, 1.95)	20	0.86	(0.44, 1.67)	205	1.01	(0.80, 1.27)	
TT	529	245	1.26	(1.04, 1.52)	37	1.20	(0.79, 1.83)	7	0.87	(0.35, 2.14)	69	1.00	(0.73, 1.36)	
P-value (raw; adjusted)			0.018, 0.11			0.39, 1.00			0.76, 1.00			0.99, 1.00		
*MMP1* (rs1144393)														
TT/TC	2811	1183	1.00		212	1.00		38	1.00		376	1.00		0.039
CC	354	113	0.71	(0.56, 0.88)	22	0.78	(0.49, 1.24)	5	1.18	(0.45, 3.10)	38	0.83	(0.58, 1.19)	
P-value (raw; adjusted)			0.003, 0.018			0.30, 1.00			0.73, 1.00			0.32, 1.00		
*MMP2* (rs243839)														
AA/AG	3018	1226	1.00		223	1.00		41	1.00		385	1.00		
GG	148	72	1.23	(0.92, 1.65)	12	1.09	(0.59, 2.00)	2	0.92	(0.22, 3.87)	30	1.54	(1.02, 2.33)	0.27
P-value (raw; adjusted)			0.17, 0.40			0.79, 0.79			0.91, 0.91			0.040, 0.20		
*MMP2* (rs1477017)														
AA	1199	464	1.00		81	1.00		16	1.00		144	1.00		0.07
AG	1478	612	1.09	(0.95, 1.26)	104	1.06	(0.79, 1.44)	18	0.86	(0.43, 1.70)	200	1.11	(0.88, 1.39)	
GG	472	219	1.24	(1.02, 1.51)	49	1.58	(1.09, 2.31)	8	1.14	(0.48, 2.70)	70	1.19	(0.88, 1.63)	
P-value (raw; adjusted)			0.030, 0.15			0.016, 0.07			0.77, 0.79			0.26, 0.52		
*MMP2* (rs243836)														
GG	921	394	1.00		88	1.00		17	1.00		129	1.00		0.06
GA/AA	2245	904	0.94	(0.81, 1.08)	147	0.69	(0.52, 0.91)	26	0.65	(0.35, 1.22)	286	0.92	(0.74, 1.15)	
P-value (raw; adjusted)			0.38, 0.40			0.008, 0.04			0.18, 0.79			0.48, 0.52		
*MMP2* (rs243845)														
CC	1307	562	1.00		105	1.00		20	1.00		194	1.00		0.14
CT	1431	582	0.93	(0.81, 1.07)	95	0.82	(0.62, 1.10)	20	0.95	(0.51, 1.78)	174	0.83	(0.67, 1.04)	
TT	428	154	0.81	(0.65, 0.99)	35	0.99	(0.66, 1.48)	3	0.50	(0.15, 1.69)	47	0.77	(0.54, 1.08)	
P-value (raw; adjusted)			0.045, 0.18			0.95, 0.95			0.26, 0.79			0.12, 0.37		
*MMP3* (rs650108)														
GG	1303	546	1.00		95	1.00		14	1.00		147	1.00		0.27
GA/AA	1863	752	1.03	(0.89, 1.18)	140	1.06	(0.80, 1.41)	29	1.39	(0.71, 2.74)	267	1.27	(1.01, 1.59)	
P-value (raw; adjusted)			0.72, 1.00			0.68, 1.00			0.34, 1.00			0.038, 0.11		
*MMP9* (rs3918261)														
AA	2452	1002	1.00		194	1.00		36	1.00		339	1.00		0.09
AG/GG	714	295	0.98	(0.84, 1.14)	41	0.71	(0.50, 1.00)	7	0.69	(0.30, 1.57)	75	0.76	(0.58, 0.99)	
P-value (raw; adjusted)			0.78, 1.00			0.05, 0.13			0.38, 0.55			0.045, 0.11		
*MMP9* (rs3918249)														0.16
TT	1465	626	1.00		112	1.00		23	1.00		210	1.00		
TC	1352	526	0.88	(0.77, 1.01)	92	0.87	(0.65, 1.16)	18	0.88	(0.47, 1.65)	170	0.88	(0.71, 1.10)	
CC	344	141	0.90	(0.72, 1.12)	28	1.01	(0.65, 1.57)	2	0.39	(0.09, 1.68)	33	0.67	(0.45, 0.99)	
P-value (raw; adjusted)			0.35, 0.85			0.95, 0.95			0.21, 0.52			0.046, 0.11		

1 Adjusted for age, study center, reference year BMI, parity and genetic admixture.

2 P values are given for raw and adjusted for multiple comparisons; includes only SNPs with significant effects.

### MMP polymorphisms and tumor stage and grade and breast cancer survival

Other tumor characteristics, i.e. stage and grade, that could indicate metastatic potential also were evaluated (data not shown in table). Only *MMP3* was significantly associated with more advanced tumor stage. The AA genotype of *MMP3* rs650108 and the GG genotype of rs522616 were associated with almost a four-fold increased risk of having a distant tumor versus a tumor staged as localized or in situ (OR 4.1, 95% CI 1.09, 15.39 and OR 3.91, 95% CI 1.04 , 14.71 respectively) relative to the major allele genotype. Evaluation of tumor grade showed that women with the *MMP1* rs17293761 TT genotype were less likely to have a poorly differentiated tumor than a well differentiated tumor (OR 0.06, 95% CI<0.01–0.46; p_adj_ 0.045).

Both *MMP1* (2 of 9 SNPs) and *MMP2* (6 of 8 SNPs) were associated with survival after adjusting for disease stage (Table 5). Only *MMP1* rs17293823 was associated with different survival patterns according to percent Native American ancestry (p interaction 0.03). Specifically, the GA/AA genotypes of rs17293823 were associated with a reduced risk of death among those with the least Native American ancestry whereas a non-significant increased risk of death was found among those with most Native American ancestry. Evaluation of haplotypes (Table S3) showed that for *MMP1* the TG and CA haplotypes of rs5854 and rs17293823 were associated with survival among those with more European ancestry, whereas two *MMP2* haplotypes were associated with survival among those with more Native American ancestry; with ACATAC significantly increasing risk of dying and its converse decreasing risk of dying (see online supplement for haplotype results). ARTP supported the independent SNP assessment with an overall pathway p value of 0.0041and for *MMP2* specifically (p_artp_ = 0.0007).

**Table 5:** Associations between *MMP* genes and survival after diagnosis with breast cancer by Native American Ancestry.

	All cases			Cases with 0–28% Native American Ancestry			Cases with 29–100% Native American Ancestry			
	Deaths/Person Years	HR^1^	(95% CI)	Deaths/Person Years	HR	(95% CI)	Deaths/Person Years	HR	(95% CI)	Interaction p value
*MMP1 (rs5854)*										0.07
CC	104/10320	1.00		46/5168	1.00		58/5152	1.00		
CT	90/8345	1.10	(0.83, 1.47)	60/5607	1.34	(0.91, 1.99)	30/2739	0.93	(0.59, 1.45)	
TT	23/1764	1.27	(0.81, 2.02)	19/1312	1.76	(1.02, 3.03)	4/452	0.66	(0.24, 1.83)	
P-value (raw; adjusted)^2^		0.30, 1.00			0.04, 0.26			0.42, 1.00		
*MMP1* (rs17293823)										0.03
GG	183/15984	1.00		106/8953	1.00		77/7031	1.00		
GA/AA	34/4432	0.73	(0.50, 1.06)	19/3134	0.53	(0.33, 0.88)	15/1299	1.25	(0.71, 2.19)	
P-value (raw; adjusted)		0.10, 0.76			0.01, 0.09			0.44, 1.00		
*MMP2* (rs1477017)										0.27
AA	95/7220	1.00		55/4642	1.00		40/2578	1.00		
AG/GG	120/13135	0.64	(0.49, 0.85)	68/7391	0.72	(0.50, 1.03)	52/5744	0.54	(0.35, 0.82)	
P-value (raw; adjusted)		0.002, 0.006			0.07, 0.26			0.004, 0.019		
*MMP2* (rs1992116)										0.65
CC	87/6597	1.00		46/3666	1.00		41/2931	1.00		
CT/TT	130/13811	0.68	(0.52, 0.89)	79/8412	0.71	(0.49, 1.03)	51/5399	0.62	(0.41, 0.95)	
P-value (raw; adjusted)		0.006, 0.017			0.07, 0.26			0.027, 0.11		
*MMP2* (rs243836)										0.95
GG	56/6339	1.00		29/3528	1.00		27/2810	1.00		
GA	103/9924	1.15	(0.83, 1.59)	63/5894	1.33	(0.86, 2.07)	40/4030	0.94	(0.58, 1.54)	
AA	58/4167	1.60	(1.10, 2.31)	33/2664	1.58	(0.96, 2.62)	25/1503	1.66	(0.95, 2.88)	
P-value (raw; adjusted)		0.013, 0.017			0.07, 0.26			0.07, 0.11		
*MMP2* (rs243845)										0.33
CC	87/9036	1.00		50/4886	1.00		37/4150	1.00		
CT	93/8904	1.14	(0.85, 1.53)	50/5426	0.91	(0.61, 1.36)	43/3478	1.51	(0.97, 2.36)	
TT	37/2490	1.70	(1.15, 2.52)	25/1774	1.59	(0.97, 2.59)	12/715	1.96	(1.01, 3.80)	
P-value (raw; adjusted)		0.008, 0.017			0.07, 0.26			0.048, 0.11		
*MMP2* (rs11639960)										0.38
AA	116/8627	1.00		63/5023	1.00		53/3603	1.00		
AG/GG	101/11803	0.59	(0.45, 0.78)	62/7064	0.65	(0.46, 0.93)	39/4739	0.51	(0.34, 0.78)	
P-value (raw; adjusted)		<.001, 0.001			0.018, 0.09			0.002, 0.01		
*MMP2* (rs11541998)										0.09
CC	186/16472	1.00		99/9352	1.00		87/7120	1.00		
CG/GG	30/3950	0.70	(0.48, 1.04)	25/2728	0.88	(0.57, 1.37)	5/1222	0.36	(0.15, 0.90)	
P-value (raw; adjusted)		0.07, 0.07			0.57, 0.57			0.028, 0.11		

1 Hazard Ratios (HR) and 95% CI adjusted for age, study center, reference year BMI, parity, genetic admixture, and SEER tumor stage.

2 P values are given for raw and adjusted for multiple comparisons; includes only SNPs with significant effects.

## Discussion

Our findings suggest that MMP genes are associated with breast cancer risk and survival after diagnosis in this genetically admixed population (Table 6 summarizes study findings). While most associations were modest, multiple polymorphisms in *MMP1* and *MMP2* were associated with breast cancer risk overall and with ER+ tumors. *MMP3* and *MMP9* were associated with breast cancer risk among those with most Native American ancestry and those with ER−/PR− tumors. Only *MMP1* and *MMP2* were associated with survival after diagnosis with breast cancer. We observed minimal differences in risk by menopausal status. Overall the *MMP* pathway was associated with both breast cancer risk and survival as indicated by a significant p value using the ARTP statistic.

**Table 6:** Summary table of significant SNP associations with breast cancer risk overall and by Native American ancestry strata, ER/PR status, and survival.

		Breast Cancer				ER/PR Status				Survival		
		All Women	0–28% NA Ancestry	29–70% NA Ancestry	71–100% NA Ancestry	ER+/PR+	ER+/PR−	ER−/PR+	ER−/PR−	All Women	0–28% NA Ancestry	29–100% NA Ancestry
Pathway ARTP p value		0.04	0.26	0.30	0.02	0.03	0.06	0.49	0.03	0.004	0.07	0.009
*MMP1*	rs5854	X^2^	x			x	x^1^				x	
	rs17293823										x	
	rs996999	x			x							
	rs7125062	x		x	x			x				
	rs470358					x						
	rs1144393	x				x^1^						
Gene ARTP p value		0.02	0.08	0.44	0.07	0.01	0.11	0.16	0.84	0.52	0.05	0.60
*MMP2*	rs243839								x			
	rs1477017					x	x			x^1^		x^1^
	rs1992116									x^1^		x
	rs243836						x^1^			x^1^		
	rs243845	x		x		x				x^1^		x
	rs11639960									x^1^	x	x^1^
	rs11541998	x										x
Gene ARTP p value		0.06	0.4	0.07	0.48	0.08	0.03	0.69	0.13	0.0007	0.04	0.002
*MMP3*	rs569444											
	rs650108				x				x			
Gene ARTP p value		1.00	0.66	0.27	0.06	0.83	0.92	0.67	0.04	0.90	0.96	0.68
*MMP9*	rs3918261						x		x			
	rs3918249								x			
	rs3787268				x							
Gene ARTP p value		0.45	0.17	0.77	0.02	0.29	0.14	0.68	0.05	0.62	0.98	0.15

1 Adjusted p value<0.05.

2 x designates one or more statistically significant observations where the unadjusted p value was <0.05; includes only those 18 SNPs that had a significant association for at least one strata.

Although other studies of the *MMP* genes have included Latina women, they have been based on few breast cancer cases. *MMP2* rs243865 (−1306C>T) was associated with significantly higher risk of breast cancer in a study of 90 breast cancer cases from Mexico (OR 2.15 95% CI 1.1,4.1), especially among women younger than 50 years of age [7]. A study in Brazil also did not find an association with this polymorphism [20]. Conflicting results for these polymorphisms also have been reported from two studies of breast cancer among Chinese women [6], [21]. Unlike the small study from Mexico [7], we did not see an increased risk of breast cancer with this polymorphism in women with more Native American ancestry who were primarily from Mexico; we also did not observe a significant increased risk with this polymorphism among pre-menopausal women. The discrepancies in the literature are unclear and could be attributed to sample sizes of the various studies or the potential modifying effects of genetic and lifestyle factors that differ in the populations studied.

Most studies of MMPs have focused on metastatic potential given the underlying biology of MMPs and cancer. Metastatic potential has been determined by evaluating tumor stage at time of diagnosis, tumor grade and histology. One study hypothesized that MMP-1 was involved in local invasion and that MMP-9 was involved in tumor growth and malignancy [22]. In that study conducted in Poland, *MMP1* was associated with node- negative breast cancer, whereas *MMP9* was associated with ER−/PR− tumors, greater lymph node involvement, and larger tumor size. However, Grieu et al. observed that the *MMP9* −1562 polymorphism was associated with better survival and ER positive tumors whereas survival associated with the *MMP2* rs243865 polymorphism was dependent on ER tumor status [5]. Liu and colleagues reviewed several studies to evaluate metastatic potential associated with *MMP* genes [3]. Defining metastatic potential based on lymph node involvement or distant metastasis at the time of diagnosis, they observed that the GG genotype of *MMP1* (−1607) was associated with over a two-fold increased risk of breast cancer metastasis especially among those with more European background. Reduced risk of breast cancer metastasis was observed for *MMP3* −1171 5A/6A polymorphism; *MMP9* −1562 was associated with increased metastatic potential; and no associations were observed for *MMP2* −1306.

In our study, both *MMP3* and *MMP9* were associated with ER−/PR− tumors. Additionally, we observed that *MMP3* polymorphisms were associated with tumor grade, with women having a much higher risk of a non-differentiated tumor if they had the rare variant of the *MMP3* polymorphisms. We observed few differences by tumor stage at diagnosis; however, stage is also associated with screening practices and could not be examined in this population. Our data suggest that both *MMP1* and *MMP2* influence survival. Two of nine *MMP1* SNPs were associated with survival and six of eight *MMP2* SNPs were associated with survival. Our data provide support for the hypothesis that *MMP* genes influence metastatic potential and survival. Utilization of the ARTP allowed us to focus on the significance of the pathway and of the genes. This was important given that multiple SNPs in several genes that were associated and the importance missed by multiple comparison adjustment that does not consider the overall gene importance when a high proportion of SNP are significant at the 0.02 or even 0.01 prior to multiple comparison adjustment.

Most of the literature on the biology of MMPs points to their role in maintaining cell integrity and their role in cancer invasion and metastasis. MMPs are proteolytic enzymes that degrade extracellular matrix and basement membrane. MMP-1 is one of the most widely expressed MMPs and degrades interstitial connective tissue. MMP-2 and MMP-9 play a key role in angiogenesis and MMP-3 is produced by connective tissue that activates other MMPs. Our findings suggest that all of the MMPs are involved in various aspects of breast cancer development and progression. While only *MMP1* was associated with tumor differentiation, *MMP3* and *MMP9* were associated with ER−/PR− tumors. *MMP1* and *MMP2* were associated with many aspects of breast cancer prognosis including unique associations with ER/PR tumor status as well as with survival. Six of the eight *MMP2s* evaluated were associated with survival independent of genetic ancestry, suggesting the importance of that gene in tumor progression and invasiveness for both NHW and Latina women with a wide range of Native American ancestry. These findings were confirmed by the ARTP analysis.

Ours is the largest study to date to report on MMP genes in an admixed population of U.S. and Mexican women with breast cancer and population-based controls. While we stratified the population to maximize our ability to examine the risk associated within strata of Native American ancestry, it should be recognized that cutpoints chosen were arbitrary based on this population distribution. However, we observed few differences in breast cancer risk by Native American ancestry, with only *MMP9* being different by ancestry group. Additionally we did observe a statistically significant pathway partp for women with the highest Native American ancestry but not for the other two groups, suggesting that this pathway is more important for women with greater Native American ancestry. One would hypothesize that given the biological role of MMPs that women with greater Native American ancestry could also have poorer survival associated with genes in this pathway. We did not detect differences in survival by ancestry. This lack of an association is most likely from the narrower range of Native American ancestry available for study given that the Mexico City sample did not have survival information. The highest Native American ancestry group used to evaluate breast cancer risk was comprised of mainly from women in Mexico City.

Whereas the populations in the U.S. had information on tumor characteristics such as ER and PR status and tumor grade and survival, this information was not available from Mexico. This limited our ability to evaluate these characteristics in as much detail by admixture since most women in the high Native American ancestry group were from Mexico. We hypothesized associations with specific genes and for some candidate SNPs that had previously been associated with cancer. However, in general we invoked a tagSNP approach to characterize genetic variation across the genes of interest. We acknowledge that associations could be spurious. Adjustment for multiple comparisons was made, although given the number of SNPs evaluated; some associations could be from chance. However, the study was one of candidate genes that were hypothesized to be associated with breast cancer development and progression and therefore too conservative interpretation of multiple comparison adjustments could lead to rejecting findings that may actually be true; thus replication of these findings in other studies is needed. Since information on the functionality of most of the SNPs examined is limited, our interpretation of findings is greatly guided by the literature on MMPs and their association with cancer in general. Additionally, we had limited power to evaluate variants with low minor allele frequency, and thus could have missed associations for both SNPs and haplotypes.

In this study of breast cancer in an admixed population of Hispanic and non-Hispanic white women, MMPs were associated both with breast cancer development and prognosis. Several polymorphisms were uniquely associated with ER and PR status of tumors, with *MMP3* and *MMP9* being associated with ER−/PR− tumors. *MMP1* and *MMP2* were associated with survival after diagnosis with breast cancer. The composite of data suggest that MMPs are associated with breast cancer progression. Replication of these findings by other large studies and work to determine the functionality of the polymorphisms examined will help determine the role of MMPs in breast cancer carcinogenesis.

## Supporting Information

Table S1 Breast cancer risk associated with *MMP* gene haplotypes for all women and by genetic admixture.
(DOCX)

Table S2 Breast cancer risk associated with *MMP* gene haplotypes and ER/PR status of tumors
(DOCX)

Table S3 Associations between survival and *MMP* gene haplotypes for all women and by Native American ancestry.
(DOCX)
