# Supplementary material for: Matrix Metalloproteinase Genes Are Associated with Breast Cancer Risk and Survival: The Breast Cancer Health Disparities Study
Source: PLoS One. 2013 May 16;8(5):e63165. doi: 10.1371/journal.pone.0063165 (PMC3655963; doi:10.1371/journal.pone.0063165)
Supplement: Table S1 — Breast cancer risk associated with MMP gene haplotypes for all women and by genetic admixture. (DOCX) [file pone.0063165.s001.docx]

Table S1. Breast cancer risk associated with *MMP* gene haplotypes for all women and by genetic admixture.

|  |  | All Women | | | | | | | 0 - 28% Native American Ancestry | | | | | | | | | | | 29 - 70% Native American Ancestry | | | | | | | | | 71 - 100% Native American Ancestry | | | | | | | | |
| --- | --- | --- | --- | --- | --- | --- | --- | --- | --- | --- | --- | --- | --- | --- | --- | --- | --- | --- | --- | --- | --- | --- | --- | --- | --- | --- | --- | --- | --- | --- | --- | --- | --- | --- | --- | --- | --- |
|  | Haplotype | Freq. | OR | (95% CI) | | | P | | Freq | | OR | | | (95% CI) | | | | P | | Freq | OR | | (95% CI) | | | | P | | Freq | OR | | | (95% CI) | | | | P |
| *MMP1* rs5854(C>T), rs996999(C>T), rs7125062(T>C), rs1144393(T>C) | | | | | | | | | | | | |  | |  | |  | |  | |  |  | |  | |  | |  | | |  |  | |  | |  | |
|  | C-C-T-T | 0.27 | 1.07 | (0.99, | 1.15) | | 0.07 | | 0.25 | | 1.14 | | | (1.02, | | 1.26) | | 0.017 | | 0.28 | 1.06 | | (0.95, | | 1.19) | | 0.32 | | 0.27 | 0.85 | | | (0.69, | | 1.04) | | 0.10 |
|  | C-T-C-T | 0.20 | 1.03 | (0.95, | 1.11) | | 0.51 | | 0.15 | | 0.99 | | | (0.87, | | 1.13) | | 0.88 | | 0.22 | 0.98 | | (0.86, | | 1.11) | | 0.72 | | 0.30 | 1.25 | | | (1.04, | | 1.51) | | 0.018 |
|  | T-C-T-T | 0.16 | 0.97 | (0.89, | 1.06) | | 0.50 | | 0.18 | | 0.97 | | | (0.86, | | 1.10) | | 0.63 | | 0.16 | 0.95 | | (0.83, | | 1.09) | | 0.49 | | 0.12 | 0.99 | | | (0.76, | | 1.30) | | 0.96 |
|  | T-C-T-C | 0.11 | 0.88 | (0.79, | 0.97) | | 0.012 | | 0.17 | | 0.88 | | | (0.77, | | 0.99) | | 0.04 | | 0.07 | 0.91 | | (0.74, | | 1.11) | | 0.34 | | 0.03 | 0.85 | | | (0.52, | | 1.39) | | 0.52 |
|  | C-C-C-T | 0.10 | 1.03 | (0.92, | 1.15) | | 0.62 | | 0.04 | | 1.01 | | | (0.80, | | 1.29) | | 0.92 | | 0.13 | 1.08 | | (0.92, | | 1.26) | | 0.34 | | 0.21 | 0.97 | | | (0.78, | | 1.21) | | 0.78 |
|  | C-C-T-C | 0.09 | 0.98 | (0.87, | 1.10) | | 0.70 | | 0.11 | | 1.01 | | | (0.87, | | 1.17) | | 0.92 | | 0.07 | 0.95 | | (0.78, | | 1.15) | | 0.58 | | 0.04 | 0.83 | | | (0.52, | | 1.33) | | 0.44 |
| *MMP2* rs243845(C>T), rs11541998(C>G) | | | | | |  | |  | | | |  |  | |  | |  | |  | |  |  | |  | |  | |  | | |  |  | |  | |  | |
|  | C-C | 0.58 | 1.03 | (0.97, | 1.10) | | 0.34 | | 0.52 | | 1.02 | | | (0.93, | | 1.12) | | 0.68 | | 0.61 | 1.06 | | (0.95, | | 1.18) | | 0.28 | | 0.73 | 1.06 | | | (0.87, | | 1.30) | | 0.55 |
|  | T-C | 0.34 | 0.93 | (0.87, | 0.99) | | 0.028 | | 0.38 | | 0.94 | | | (0.85, | | 1.03) | | 0.21 | | 0.32 | 0.91 | | (0.81, | | 1.01) | | 0.08 | | 0.25 | 0.92 | | | (0.75, | | 1.14) | | 0.45 |
|  | C-G | 0.08 | 1.13 | (1.00, | 1.27) | | 0.045 | | | 0.11 | 1.09 | | | (0.94, | | 1.27) | | 0.26 | | 0.07 | 1.13 | | (0.93, | | 1.38) | | 0.21 | | 0.02 | 1.13 | | | (0.63, | | 2.03) | | 0.69 |
| ^1^Odds Ratios (OR) and 95% Confidence Intervals (CI) adjusted for age, study center, reference year BMI, parity, and genetic admixture. | | | | | | | | | | | | | | | | | | | | | | | | | | | |  | | |  |  | |  | |  | |
