# Supplementary material for: Matrix Metalloproteinase Genes Are Associated with Breast Cancer Risk and Survival: The Breast Cancer Health Disparities Study
Source: PLoS One. 2013 May 16;8(5):e63165. doi: 10.1371/journal.pone.0063165 (PMC3655963; doi:10.1371/journal.pone.0063165)
Supplement: Table S2 — Breast cancer risk associated with MMP gene haplotypes and ER/PR status of tumors (DOCX) [file pone.0063165.s002.docx]

Table S2. Breast cancer risk associated with *MMP* gene haplotypes and ER/PR status of tumors

|  |  | ER+/PR+ | | | |  | ER+/PR- | | | | ER-/PR+ | | |  | ER-/PR- | | | |
| --- | --- | --- | --- | --- | --- | --- | --- | --- | --- | --- | --- | --- | --- | --- | --- | --- | --- | --- |
|  | Haplotype | Freq | OR | (95% CI) | | p | OR | (95% CI) | | p | OR | (95% CI) | | p | OR | (95% CI) | | p |
| *MMP1* rs5854(C>T), rs7125062(T>C), rs470358(C>T), rs1144393(T>C) | | | | | | | | | |  |  |  |  |  |  |  |  |  |
|  | C-C-T-T | 0.18 | 1.10 | (0.97, | 1.25) | 0.13 | 0.97 | (0.75, | 1.26) | 0.82 | 0.62 | (0.32, | 1.22) | 0.16 | 0.95 | (0.78, | 1.16) | 0.61 |
|  | T-T-C-T | 0.16 | 0.94 | (0.83, | 1.06) | 0.33 | 0.94 | (0.73, | 1.21) | 0.63 | 1.21 | (0.71, | 2.07) | 0.48 | 1.00 | (0.83, | 1.21) | 0.99 |
|  | C-T-T-T | 0.14 | 1.12 | (0.98, | 1.27) | 0.11 | 1.26 | (0.97, | 1.64) | 0.08 | 1.35 | (0.77, | 2.36) | 0.30 | 1.08 | (0.87, | 1.33) | 0.49 |
|  | C-T-C-T | 0.13 | 1.04 | (0.91, | 1.18) | 0.61 | 1.22 | (0.94, | 1.58) | 0.13 | 1.02 | (0.54, | 1.91) | 0.96 | 1.03 | (0.83, | 1.27) | 0.80 |
|  | T-T-C-C | 0.12 | 0.84 | (0.73, | 0.97) | 0.014 | 0.66 | (0.48, | 0.91) | 0.011 | 0.98 | (0.51, | 1.88) | 0.95 | 0.91 | (0.72, | 1.13) | 0.39 |
|  | C-C-C-T | 0.10 | 1.01 | (0.85, | 1.20) | 0.90 | 0.91 | (0.63, | 1.31) | 0.60 | 0.61 | (0.25, | 1.50) | 0.28 | 1.17 | (0.92, | 1.51) | 0.21 |
|  | C-C-T-C | 0.07 | 1.00 | (0.84, | 1.18) | 0.99 | 1.02 | (0.72, | 1.44) | 0.91 | 0.74 | (0.30, | 1.86) | 0.53 | 1.05 | (0.80, | 1.37) | 0.74 |
|  | C-T-C-C | 0.05 | 0.96 | (0.78, | 1.17) | 0.65 | 1.00 | (0.66, | 1.49) | 0.98 | 1.66 | (0.76, | 3.60) | 0.20 | 0.86 | (0.61, | 1.20) | 0.37 |
| *MMP2* rs243839(A>G), rs1477017(A>G), rs243836(G>A), rs243845(C>T) | | | | | | | | | |  |  |  |  |  |  |  |  |  |
|  | A-G-G-C | 0.35 | 1.1 | (1.00, | 1.22) | 0.046 | 1.25 | (1.03, | 1.52) | 0.022 | 1.11 | (0.71, | 1.74) | 0.63 | 1.12 | (0.96, | 1.30) | 0.16 |
|  | A-A-A-T | 0.27 | 0.93 | (0.84, | 1.03) | 0.19 | 0.87 | (0.70, | 1.08) | 0.21 | 0.84 | (0.50, | 1.39) | 0.49 | 0.91 | (0.77, | 1.08) | 0.28 |
|  | G-A-A-C | 0.16 | 0.98 | (0.87, | 1.12) | 0.82 | 0.91 | (0.70, | 1.19) | 0.49 | 0.88 | (0.47, | 1.63) | 0.68 | 1.12 | (0.92, | 1.36) | 0.24 |
|  | A-A-G-T | 0.06 | 0.85 | (0.71, | 1.03) | 0.10 | 1.14 | (0.81, | 1.60) | 0.47 | 1.02 | (0.44, | 2.34) | 0.97 | 0.77 | (0.57, | 1.06) | 0.11 |
|  | A-A-G-C | 0.06 | 0.93 | (0.78, | 1.12) | 0.45 | 0.76 | (0.50, | 1.15) | 0.20 | 1.54 | (0.73, | 3.23) | 0.25 | 0.85 | (0.63, | 1.16) | 0.30 |
| *MMP9* rs3918261(A>G), rs3918249(T>C) | | | | | |  |  |  |  |  |  |  |  |  |  |  |  |  |
|  | A-T | 0.70 | 1.08 | (0.98, | 1.20) | 0.12 | 1.02 | (0.83, | 1.24) | 0.87 | 1.27 | (0.78, | 2.05) | 0.34 | 1.18 | (1.00, | 1.38) | 0.048 |
|  | A-C | 0.19 | 0.92 | (0.82, | 1.03) | 0.17 | 1.14 | (0.91, | 1.43) | 0.25 | 0.86 | (0.49, | 1.51) | 0.59 | 0.96 | (0.80, | 1.15) | 0.67 |
|  | G-C | 0.10 | 0.96 | (0.83, | 1.11) | 0.60 | 0.71 | (0.51, | 0.99) | 0.044 | 0.67 | (0.31, | 1.46) | 0.32 | 0.73 | (0.57, | 0.94) | 0.016 |
|  | G-T | <.01 | 0.84 | (0.23, | 3.13) | 0.80 | 5.02 | (1.33, | 18.93) | 0.02 | 6.90 | (0.84, | 56.76) | 0.07 | 1.58 | (0.34, | 7.37) | 0.56 |
| Adjusted for age, study center, reference year BMI, parity and genetic admixture. | | | | | | | | | | |  |  |  |  |  |  |  |  |
