# Supplementary material for: Matrix Metalloproteinase Genes Are Associated with Breast Cancer Risk and Survival: The Breast Cancer Health Disparities Study
Source: PLoS One. 2013 May 16;8(5):e63165. doi: 10.1371/journal.pone.0063165 (PMC3655963; doi:10.1371/journal.pone.0063165)
Supplement: Table S3 — Associations between survival and MMP gene haplotypes for all women and by Native American ancestry. (DOCX) [file pone.0063165.s003.docx]

Table S3. Associations between survival and *MMP* gene haplotypes for all women and by Native American ancestry.

|  |  | All Women | | | | | | | | | | 0 - 28% Native American Ancestry | | | | | | | | | | | | | | | 29 - 100% Native American Ancestry | | | | | | | | | | | | | | | | | | |  |  |  |  |  |
| --- | --- | --- | --- | --- | --- | --- | --- | --- | --- | --- | --- | --- | --- | --- | --- | --- | --- | --- | --- | --- | --- | --- | --- | --- | --- | --- | --- | --- | --- | --- | --- | --- | --- | --- | --- | --- | --- | --- | --- | --- | --- | --- | --- | --- | --- | --- | --- | --- | --- | --- |
|  | Haplotype | | Frequency | | HR | | (95% CI) | | | | Wald P | | Frequency | | | HR | | | | (95% CI) | | | | | Wald P | | | | Frequency | | | HR | | | (95% CI) | | | | | | Wald P | | | | | | | |  |  |
| *MMP1* rs5854(C>T), rs17293823(G>A) | | | | | | | |  | |  | | |  | |  | | | |  | |  | |  | | | | |  | |  | | |  | | | |  | | |  | | | | | | | | | |  |
|  | C-G | | 0.59 | 1.01 | | (0.83, | | | 1.23) | | 0.90 | | | 0.52 | | | 0.97 | (0.76, | | | | 1.25) | | 0.84 | | 0.69 | | | | | 1.03 | | | | | (0.76, | | | 1.42) | | | | 0.84 | | | |  |  |  |  |
|  | T-G | | 0.29 | 1.12 | | (0.91, | | | 1.37) | | 0.29 | | | 0.34 | | | 1.33 | (1.03, | | | | 1.72) | | 0.032 | | 0.22 | | | | | 0.87 | | | | | (0.61, | | | 1.24) | | | | 0.43 | | | |  |  |  |  |
|  | C-A | | 0.12 | 0.74 | | (0.53, | | | 1.04) | | 0.08 | | | 0.14 | | | 0.53 | (0.33, | | | | 0.85) | | 0.008 | | 0.09 | | | | | 1.24 | | | | | (0.77, | | | 1.99) | | | | 0.39 | | | |  |  |  |  |
| *MMP2* rs1477017(A>G), rs1992116(C>T), rs243836(G>A), rs243845(C>T), rs11639960(A>G), rs11541998(C>G) | | | | | | | | | | | | | | | | | | | | | | | | | | | | | | | | | | | | | | | | | | | | | | |  |  |  |  |
|  | A-C-A-T-A-C | | 0.27 | 1.39 | | (1.13, | | | 1.70) | | 0.002 | | | 0.30 | | | 1.23 | (0.94, | | | | 1.61) | | | 0.14 | 0.22 | | | | 1.68 | | | | (1.22, | | | | 2.30) | | | | 0.001 | | | | |  |  |  |  |
|  | G-T-G-C-G-C | | 0.22 | 0.77 | | (0.60, | | | 0.99) | | 0.043 | | | 0.21 | | | 0.75 | (0.53, | | | | 1.05) | | | 0.10 | 0.22 | | | | 0.78 | | | | (0.54, | | | | 1.13) | | | | 0.20 | | | | |  |  |  |  |
|  | A-C-A-C-A-C | | 0.16 | 1.02 | | (0.78, | | | 1.32) | | 0.89 | | | 0.15 | | | 1.14 | (0.81, | | | | 1.61) | | | 0.45 | 0.17 | | | | 0.88 | | | | (0.59, | | | | 1.33) | | | | 0.55 | | | | |  |  |  |  |
|  | G-T-G-C-G-G | | 0.09 | 0.72 | | (0.49, | | | 1.06) | | 0.09 | | | 0.11 | | | 0.90 | (0.59, | | | | 1.40) | | | 0.65 | 0.07 | | | | 0.38 | | | | (0.16, | | | | 0.93) | | | | 0.035 | | | | |  |  |  |  |
|  | A-C-G-T-A-C | | 0.06 | 0.93 | | (0.62, | | | 1.40) | | 0.74 | | | 0.06 | | | 1.00 | (0.60, | | | | 1.67) | | | 0.99 | 0.06 | | | | 0.86 | | | | (0.43, | | | | 1.71) | | | | 0.67 | | | | |  |  |  |  |
|  | G-T-G-C-A-C | | 0.05 | 1.45 | | (0.96, | | | 2.19) | | 0.08 | | | 0.03 | | | 1.36 | (0.69, | | | | 2.68) | | | 0.38 | 0.07 | | | | 1.52 | | | | (0.91, | | | | 2.56) | | | | 0.11 | | | | |  |  |  |  |
|  | A-T-G-C-A-C | | 0.05 | 0.96 | | (0.62, | | | 1.50) | | 0.86 | | | 0.07 | | | 0.96 | (0.58, | | | | 1.60) | | | 0.89 | 0.03 | | | | 0.92 | | | | (0.37, | | | | 2.32) | | | | 0.86 | | | | |  |  |  |  |
|  | A-C-G-C-A-C | | 0.03 | 1.36 | | (0.84, | | | 2.19) | | 0.21 | | | 0.02 | | | 1.19 | (0.45, | | | | 3.15) | | | 0.73 | 0.05 | | | | 1.45 | | | | (0.84, | | | | 2.53) | | | | 0.18 | | | | |  |  |  |  |
| ^1^Hazard Ratios (HR) and 95% Confidence Intervals (CI) adjusted for age, study center, reference year BMI, parity, genetic admixture, and SEER tumor stage. | | | | | | | | | | | | | | | | | | | | | | | | | | | | | | | | | | | | | | | | | | | |  |  | | |  |  |  |
